# Patients’ willingness and needs for ICU virtual reality visits before elective surgery: a qualitative study

**DOI:** 10.3389/fpubh.2026.1902729

**Published:** 2026-09-09

**Authors:** Chunmei Fan, Jicheng Zhang, Nan Cheng, Zhengang Wei, Zhenfeng Zhou, Yingxue Sun, Heng Cao, Fangyan Yue, Congcong Liu

**Affiliations:** Intensive Care Unit, Shandong Provincial Hospital Affiliated to Shandong First Medical University, Jinan, Shandong, China

**Keywords:** intensive care unit, patient education, preoperative visit, qualitative study, virtual reality

## Abstract

**Objective:**

To investigate patients’ willingness and needs for ICU virtual reality visits prior to elective surgery, so as to provide a reference for developing VR-based preoperative ICU visits tools that meet actual clinical user requirements.

**Methods:**

A descriptive qualitative study was conducted. A total of 18 patients undergoing elective surgery in surgical departments collaborating with the intensive care unit of a tertiary Grade A hospital in Jinan, Shandong Province, between March 2025 and August 2025 were selected for semi-structured in-depth interviews. Data were analyzed using thematic analysis.

**Results:**

Five themes were extracted: current cognitive status of patients regarding the ICU and core information gaps; patients’ cognition and expectations of preoperative visits; patients’ acceptance and cognition of applying VR technology to ICU preoperative visits; core needs for VR-based preoperative ICU visits; and Potential Barriers and Optimization Suggestions for the Implementation of VR-based preoperative ICU visits.

**Conclusion:**

This qualitative study explored patients’ willingness and needs for VR-based preoperative ICU visits. Patients expressed positive attitudes toward the potential application of VR technology in ICU preoperative visits, reflecting anticipated rather than actual use of this intervention. Based on the results of this study, future research can collaborate with technology development teams to construct a VR-based preoperative ICU visits system, conduct longitudinal in-depth studies from the aspects of clinical verification, technical iteration, and long-term promotion, so as to facilitate the translation of this technology from demand-oriented design to clinical application.

## 1 Introduction

As an essential component of perioperative care, preoperative visits are crucial for patients’ physical and psychological preparation and exert a positive impact on the entire anesthesia and surgical process. Its core value lies in alleviating preoperative anxiety and improving treatment compliance through precise information delivery and psychological intervention (1). Providing information helps patients understand what will occur during hospitalization and treatment, enhances treatment adherence, and minimizes their worries and anxiety (2). Conventionally, operating room nurses conduct preoperative visits to deliver necessary information and education regarding surgical procedures and related care, strengthen patients’ trust in the surgical team, reduce their fear of the surgical environment, allow patients to express concerns and fears about the upcoming surgery, and facilitate postoperative recovery (1).

In China, for elective surgical patients with complex conditions, extensive surgical trauma, or comorbidities, postoperative admission to the intensive care unit (ICU) for intensive treatment and monitoring has become an important medical strategy to improve prognosis. However, such patients often experience psychological stress such as uncertainty about and fear of the ICU preoperatively, leading to an increased incidence of preoperative anxiety (3, 4). Preoperative anxiety has been confirmed to be closely associated with postoperative delirium, prolonged mechanical ventilation, extended length of hospital stay, and increased medical costs (5–8). Traditional preoperative education mostly relies on verbal explanation, printed brochures, or two-dimensional videos, which are limited by abstract information transmission and insufficient interactivity. Although some studies have attempted to improve patients’ cognition through in-person ICU visits (9), large-scale implementation remains challenging due to constraints such as infection control requirements and clinical workflow limitations (10).

Recent studies have explored the potential of virtual reality (VR) in patient education and demonstrated that VR is effective in enhancing clinical knowledge, improving patients’ understanding of procedures, and reducing anxiety across various clinical settings (11, 12). In the perioperative period, compared with standard verbal and written information, VR-based patient education videos provide superior informational and procedural knowledge, reduce preoperative worry and anxiety, and enhance patient satisfaction following surgery (13). VR creates a simulated environment in which users interact with computer-generated visuals and sounds using devices such as headsets and motion sensors (14–16), improving patients’ comprehension of three-dimensional (3D) structures and establishing an immersive environment that promotes focused engagement with the presented content (11). Therefore, with its unique advantages of immersive scenario construction and intuitive information presentation, VR technology offers a novel approach to overcoming the limitations of traditional preoperative visits.

However, notable gaps remain in the application of VR technology to preoperative visits. First, there is a narrow focus of scenarios: existing studies predominantly center on the display of operating room environments and simulation of routine surgical procedures (17–19), with a severe scarcity of VR visit tools targeting the specific setting of the ICU. Second, content adaptation is insufficient: most current ICU-related VR programs for patients are commercially available generic versions, lacking customized design aligned with the core needs of critically ill patients. Such programs often differ from actual clinical practice in terms of medical workflows and equipment configurations, limiting their direct applicability. Third, a patient-centered development logic is absent. The Virtual Reality Clinical Outcomes Research Experts Model, a widely adopted guideline for developing and testing therapeutic VR systems, divides VR clinical research into three progressive phases: system content design, early system testing, and randomized controlled trial, and explicitly mandates end user participation in intervention development during the system content design phase (20). Most patient facing VR programs do not involve patients in the development process, violating this core principle and resulting in inadequate practicality and adaptability of the resulting tools.

VR-based preoperative ICU visits specifically refer to preoperative interventions using VR technology for patients scheduled for elective surgery who are expected to be admitted to the ICU postoperatively. Their core value is to proactively fill patients’ knowledge gaps regarding the ICU, alleviate preoperative psychological stress, and lay the foundation for a smooth transition to the ICU environment after surgery. To date, no studies in China have reported the development of VR-based preoperative ICU visits tools for patients anticipating ICU admission following surgery, and systematic exploration of patients’ willingness and core needs for such tools remains lacking.

The present study is positioned at the development phase of this framework, which is applied exclusively as a procedural roadmap to guide our VR-based preoperative ICU visits system development. Importantly, this framework was not used as a predefined coding framework for qualitative data analysis; all themes were generated through a fully inductive analytical process. Adopting a qualitative approach, we explore in depth patients’ perceptions, willingness, and core needs regarding VR-based preoperative ICU visits, aiming to provide a scientific basis for developing standardized, user-centered VR educational tools for ICU preoperative care.

## 2 Participants and methods

### 2.1 Participants

This study was conducted in a closed general ICU of a tertiary Grade A hospital in Jinan, China. Routine preoperative care includes only general verbal education without dedicated ICU orientation, and in-person ICU visits are unavailable due to infection control policies.

A purposive sampling method was used to select patients undergoing elective surgery in surgical departments collaborating with the ICU of a tertiary Grade A hospital in Jinan, Shandong Province, from March 2025 to August 2025, who were expected to be admitted to the ICU postoperatively. Inclusion criteria: ① Patients scheduled for elective surgery with planned postoperative ICU admission (conscious and with normal communication ability); ② Age ≥ 18 years; ③ Informed consent and willingness to participate in interviews. Exclusion criteria: ① Patients with severe cognitive impairment or mental illness; ② Those unable to cooperate with the interview. A maximum variation sampling strategy was implemented prospectively to enhance the comprehensiveness of the findings. Heterogeneity was intentionally targeted across five predefined dimensions: age, educational level, surgical type, prior ICU admission experience, and familiarity with VR technology. Throughout the recruitment period, we monitored the composition of the enrolled sample and adjusted recruitment priorities to ensure representation across all targeted dimensions.

A total of 18 patients were interviewed in this study, labeled with “P” for “Patient” in the text. Among them, 10 were male and 8 female, aged 38–72 years (mean ± SD: 55.86 ± 11.02 years). Eight had a junior high school education or below, 6 had a senior high school or technical secondary school education, and 4 had a bachelor’s degree or above. Two patients had heard of VR, and 2 had previously used VR, mainly for entertainment and sports. Surgical types included abdominal (*n* = 6), cardiothoracic (*n* = 3), orthopedic surgery (*n* = 5), and other types of surgery (*n* = 4).

### 2.2 Methods

#### 2.2.1 Development of the interview guide

A preliminary interview guide was developed through group discussion by the research team. A pilot interview was conducted with 2 patients to assess the feasibility of the formal interview. The guide was then revised and refined based on feedback from the pilot interview, and the final version was established (see Table 1 for details).

**Table 1:** Interview guide for patients.

No.	Interview questions
1	How much do you know about the ICU? Through which channels have you obtained relevant information?
2	Have you ever heard of preoperative visits before? If yes, what do you think is the significance of preoperative visits for you?
3	Have you ever heard of or used any virtual reality (VR) systems or devices before? If you have used them, what was the purpose of your use, and what functions did they have?
4	What do you think of using virtual reality technology for ICU preoperative visits? What functions do you hope such a system can have? In your opinion, what kind of knowledge content and presentation methods can better meet your needs?
5	What are your views on the feasibility and potential value of applying virtual reality technology to ICU preoperative visits?
6	Would you be willing to receive preoperative visits via a virtual reality system? Why?
7	During the use of the ICU virtual reality visit, what barriers do you think you may face? How can these barriers be solved?
8	Do you have any additional suggestions for the design and application of the ICU preoperative virtual reality visit system? What are your views on its future development?

#### 2.2.2 Data collection

A descriptive qualitative design was used, with semi-structured face-to-face interviews. Interviews were conducted in a quiet, comfortable, private, and undisturbed meeting room or consultation room. The lead researcher conducting the interviews was a Master of Nursing with systematic training in qualitative research and prior experience in qualitative interview studies. The interviewer had no prior professional or personal relationship with any participants and was not involved in their clinical care, to minimize response bias related to clinical role dynamics. To uphold reflexivity, the researcher adopted a bracketing approach throughout the study: prior assumptions about VR-based preoperative ICU visits and patients’ psychological needs were actively documented and set aside, to prevent pre-existing perspectives from influencing data collection and interpretation (Table 2).

**Table 2:** Thematic coding examples.

Theme	Subtheme	Initial codes	Interview transcripts
3.1. Theme 1: Current Cognitive Status of Patients Regarding the ICU and Core Information Gaps	3.1.1. Diversity and Limitations of Information Sources	Information from previous personal ICU experience	P3: “I stayed in the ICU before and felt lonely and scared; I still worry about it now.” P7: “The ICU more than 10 years ago must be different from now. I want to know the latest situation.”
		Subjective Information from relatives/friends/fellow patients	P1: “Relatives told me that once you go into the ICU, it’s hard to get out.”
		Information from the Internet	P2: “Online sources say the ICU environment is depressing and family members are not allowed in, but I have no idea what it’s really like inside.”
		Insufficient information from doctors	P8: “The doctor only said I would be admitted to the ICU for observation after surgery but did not explain what would be done there.”
	3.1.2. Core Cognitive Gaps	Lack of knowledge about ICU physical environment and layout	P4: “I do not know what the ICU looks like and have no understanding of its environment and layout.”
		Unfamiliarity with post-operative treatment and nursing workflows	P6: “After I enter the ICU, how often will the doctor check on me and what treatments will I receive?”
		Poor understanding of core equipment functions and alarm meanings	P17: “I do not know what the numbers on the monitor mean. Does an alarm mean my condition is getting worse?”
		Unclear criteria for ICU discharge	P5: “I want to know when I can be transferred out of the ICU.”

Before the interview, the researcher fully explained the purpose and significance of the study, introduced the interview content and procedure, and provided basic explanations of VR-based preoperative ICU visits to ensure participants had a fundamental understanding of the technology. During the interview, the researcher listened carefully, asked follow-up questions to clarify ambiguous statements, and maintained a neutral attitude toward participants’ views. The content and sequence of questions were adjusted flexibly to elicit in-depth information. Nonverbal cues such as meaningful facial expressions, tone, and body language were also documented.

All interview content was kept confidential and would be destroyed promptly upon completion of the study. Each interview lasted 20–45 min and was audio-recorded in its entirety. The studies involving human participants were reviewed and approved by the Medical Ethics Committee of Shandong Provincial Hospital Affiliated to Shandong First Medical University (approval no. 2025–929). The studies were conducted in accordance with the local legislation and institutional requirements. The participants provided their written informed consent to participate in this study.

#### 2.2.3 Data processing and analysis

Audio recordings were transcribed verbatim into text by the researcher within 24 h after each interview. Two researchers (the primary researcher and another postgraduate student) independently imported the transcripts into NVivo 14.0 software for analysis. Aligned with the core tenets of Braun and Clarke’s reflexive thematic analysis, this independent double coding practice was adopted as a complementary rigor enhancing strategy rather than a formal inter coder reliability measure. It was designed to mitigate individual researcher bias and deepen interpretive reflexivity through collaborative, data-centered discussion, consistent with the reflexive approach’s emphasis on transparent, contextualized interpretation. Data were analyzed using the 6-phase thematic analysis method by Braun and Clarke (21): ① Familiarization with data through repeated reading of transcripts; ② Initial coding; ③ Searching for themes; ④ Reviewing themes; ⑤ Defining and naming themes; ⑥ Producing the final report. Coding disagreements were resolved through a structured tiered process. First, the two coders revisited the original interview transcripts to re-examine the full contextual meaning of the relevant data and discussed discrepancies to reach consensus. Any remaining unresolved differences were arbitrated by a third senior qualitative researcher with specialized experience in reflexive thematic analysis. All final themes were collectively reviewed and validated by the full research team to ensure analytical coherence. Data saturation was monitored prospectively throughout the entire data collection process: each interview was transcribed and coded immediately after completion, and the codebook was updated iteratively to track the emergence of new concepts. Data saturation was confirmed when no new codes or distinct thematic insights emerged in three consecutive interviews. Saturation was formally reached at the 16th interview, and the remaining 2 interviews were conducted to further validate saturation stability. This saturation assessment was framed to align with the reflexive approach, focusing on the conceptual depth of themes rather than rigid statistical reliability metrics. Given the descriptive qualitative design with a focused semi-structured interview guide and maximum variation sampling, this criterion is methodologically appropriate to ensure sufficient analytical depth without redundant data collection.

#### 2.2.4 Trustworthiness

Methodological rigor was established following the four trustworthiness criteria proposed by Lincoln and Guba (22): Credibility: Independent double coding with a tiered disagreement resolution process, member checking with 5 participants, and data saturation were adopted to ensure authentic findings. Dependability: A complete audit trail (including interview guide revisions, verbatim transcripts, codebooks, and theme evolution records) was maintained to enable full reconstruction of the analytical pathway. Confirmability: Reflexive memos were kept throughout the process to document analytical decisions and mitigate researcher bias. All themes were anchored in raw data and collectively validated by the research team. Transferability: Thick description of the study context and participant characteristics is provided for readers to assess the applicability of findings to other settings.

## 3 Results

### 3.1 Theme 1: current cognitive status of patients regarding the ICU and Core information gaps

#### 3.1.1 Diversity and limitations of information sources

The 18 patients obtained information about the ICU from diverse sources, characterized by fragmented and inaccurate content. Sources included experiences shared by relatives, friends, or fellow patients (often mixed with subjective personal feelings), media or online information, verbal explanations from medical staff, and previous personal ICU experience.

*P3: “I stayed in the ICU before and felt lonely and scared; I still worry about it now.”*

*P1: “Relatives told me that once you go into the ICU, it’s hard to get out.”*

*P2: “Online sources say the ICU environment is depressing and family members are not allowed in, but I have no idea what it’s really like inside.”*

*P8: “The doctor only said I would be admitted to the ICU for observation after surgery but didn’t explain what would be done there.”*

*P7: “The ICU more than ten years ago must be different from now. I want to know the latest situation.”*

#### 3.1.2 Core cognitive gaps

Patients’ knowledge gaps regarding the ICU mainly focused on: the physical environment and layout of the ICU; specific treatments, procedures, and nursing workflows after transfer from the operating room to the ICU; lack of understanding of the functions, indications, and alarm meanings of core equipment such as ventilators and monitors; and clear criteria for ICU discharge.

*P4: “I don’t know what the ICU looks like and have no understanding of its environment and layout.”*

*P6: “After I enter the ICU, how often will the doctor check on me and what treatments will I receive?”*

*P17: “I don’t know what the numbers on the monitor mean. Does an alarm mean my condition is getting worse?”*

*P5: “I want to know when I can be transferred out of the ICU.”*

### 3.2 Theme 2: patients’ cognition and expectations of preoperative visits

#### 3.2.1 Evaluation of traditional preoperative visits

Patients generally acknowledged the necessity of existing preoperative visits but also identified limitations, including inefficient information delivery, insufficient targeted content, excessive professional jargon, complicated explanations, and reliance on verbal instruction leading to poor retention.

*P1: “I couldn’t understand some professional terms the nurse used, and I can’t remember so many precautions.”*

*P12: “The nurse mainly talked about fasting before surgery but didn’t mention anything about the ICU.”*

*P9: “I don’t have a medical background, so it’s hard for me to understand medical information just by listening to medical staff.”*

#### 3.2.2 Core expectations for preoperative visits

Patients’ expectations centered on reducing fear of the unknown and relieving preoperative anxiety through early access to ICU-related information.

*P6: “If I could know about the ICU in advance, I wouldn’t lie awake all night.”*

*P7: “If I know why my hands are restrained and tubes are inserted, I will cooperate with the nurses.”*

### 3.3 Theme 3: patients’ acceptance and perceptions of applying VR technology to ICU preoperative visits

#### 3.3.1 Level of awareness of VR technology

Patients showed considerable variation in awareness of VR. Most had never heard of or used VR and had little understanding of its functions. Only 2 patients had heard of VR and recognized its immersive feature, and 2 had prior experience using VR devices for entertainment or sports with basic operational knowledge.

*P1: “What is VR?”*

*P2: “I know VR can be used to watch 3D movies; I didn’t expect it to be used in medical care.”*

*P14: “I have watched movies with VR before. Wearing the glasses lets you see virtual scenes, which are quite realistic.”*

#### 3.3.2 Acceptance and motivating factors

All patients expressed willingness to accept VR-based preoperative ICU visits. Motivating factors included the belief that visual demonstrations were easier to understand and remember than verbal explanations, and the expectation of reducing fear through early familiarization with the ICU environment and procedures.

*P3: “Just listening to the nurse isn’t enough; I need to see the ICU with my own eyes to feel relieved.”*

*P7: “If I had seen the ICU in advance, I wouldn’t be scared when I wake up with a tube in my mouth.”*

Some patients also expressed curiosity and novelty toward VR and hoped to receive condition-specific personalized information.

*P2: “VR sounds amazing; I want to experience it.”*

*P12: “I hope VR can show the postoperative nursing process for my complex surgery.”*

#### 3.3.3 Potential concerns

Some older adult and low-education patients worried about being unable to operate VR devices.

*P4: “I can’t even use a smartphone well; I’m afraid I won’t learn how to use VR glasses.”*

Others expressed concern about dizziness, nausea, or other discomfort when wearing VR headsets.

*P5: “I heard wearing VR glasses causes dizziness. I have high blood pressure and worry I can’t tolerate it.”*

Two patients feared excessive or complicated VR content would be difficult to remember.

*P17: “I hope the content is concise and clear, without too much irrelevant information, otherwise I won’t remember it.”*

### 3.4 Theme 4: Core needs for VR-based preoperative ICU visits

#### 3.4.1 Practical and personalized content focus

Patients’ content demands for VR-based preoperative ICU visits were strongly practical and personalized. Core content needs included: ICU environment and layout, including ward structure, equipment placement, and nurse station location.

*P18: “I want to see what the ICU beds look like and what instruments are around.”*

Treatment and nursing workflows covering the full process from ICU admission after surgery to ward rounds and nursing care.

*P3: “I want to know what the nurse will do first after I arrive in the ICU and how often the doctor does rounds.”*

Functions and purposes of key equipment, especially ventilators and monitors.

*P5: “I want to know what the numbers on the monitor mean and how the ventilator helps me breathe.”*

Emergency response and communication, including how to call for nurses and contact family members.

*P4: “I want to know how to call a nurse if I feel uncomfortable and whether I can contact my family.”*

#### 3.4.2 Intuitive and convenient presentation

Patients emphasized intuitive, concise, and easy-to-operate presentation formats, including minimal text, visual scenes combined with narration, moderate speaking speed, and plain language.

*P5: “Use as little text as possible. Show more scenes and animations with narration, and speak slowly.”*

*P16: “Keep the operation simple; don’t make it too complicated.”*

#### 3.4.3 Interactive and adaptive functions

Patients’ functional requirements for the VR-based preoperative ICU visits system mainly included: Interactive query functions, such as accessing detailed explanations by clicking on equipment or process steps

*P5: “When I see a ventilator, can I click on it to get a detailed introduction?”*

Personalized adjustment functions, such as narration speed, subtitle toggle, and screen brightness.

*P4: “Can we adjust the speaking speed? I have poor hearing and would like it slower.”*

Accompanied viewing and real-time Q&A, allowing nurses or family members to stay nearby and answer questions.

*P1: “I hope a nurse is beside me to teach me how to use it and answer questions whenever I don’t understand something.”*

Multi-device compatibility supporting VR headsets, mobile phones, and other platforms.

*P18: “If I don’t have VR glasses or feel dizzy wearing them, can I watch a simplified version on my phone?”*

### 3.5 Theme 5: potential barriers and optimization suggestions for the implementation of VR-based preoperative ICU visits

#### 3.5.1 Potential barriers

In this study, the perceived barriers to the implementation of VR-based preoperative ICU visits by patients mainly focused on four dimensions: equipment, content, patients themselves, and environment, as detailed below:

Equipment-related barriers: Patients were primarily concerned about the operational difficulty, wearing comfort, and hygiene safety of VR devices, especially the resistance of groups with low operational ability to the complexity of the equipment.

*P1: “I can’t even use a smartphone well, let alone this* real-time *glasses. I don’t know how to operate it.”*

*P3: “I felt dizzy when I wore VR glasses before; I definitely can’t stand wearing them for a long time,” highlighting the problem of insufficient wearing comfort (dizziness, weight-bearing).*

*P9: “So many people use these glasses; will they be unclean? It would be troublesome if they spread germs.”*

Content-related barriers: Patients’ core demands focused on the personalized adaptation of content and the control of information load.

*P5: “If the content in VR is the same for all patients, regardless of the type of surgery, then for a patient like me who undergoes hepatobiliary surgery, much of the information will be useless, and the important points will not be covered.”*

*P17: “I hope the content is not too complicated. If too many instruments and processes are explained at once, I won’t remember them, and it will only make me more anxious.”*

Patient-related barriers: Such barriers were mainly related to the physiological functions and cognitive characteristics of older adult patients. Some patients had a resistant attitude toward high-tech equipment.

*P4: “I’m 72 years old now, and I learn new things slowly. Even if the nurse teaches me to use VR, I may not learn it.”*

*P15: “I’ve never touched this kind of glasses before; I’m really afraid I won’t learn how to use them at that time.”*

Environment-related barriers: Patients paid attention to the adaptability of the experience environment and the accessibility of support resources, such as the lack of a quiet experience environment and insufficient nurse guidance time.

*P16: “The ward is crowded and noisy. If I experience VR in the ward, I definitely won’t be able to concentrate on watching.”*

*P8: “Nurses are usually very busy. If I encounter problems during the experience, I may not find a nurse to answer them in time, which will make me more anxious.”*

#### 3.5.2 Optimization suggestions

In response to the above potential barriers, patients put forward targeted optimization suggestions from three dimensions: equipment adaptation, content customization, and support system, as detailed below:

Equipment optimization: Focus on simplifying equipment operation, improving wearing comfort, and ensuring hygiene.

*P1: “Can there be fewer operation buttons?” to lower the operational threshold.*

*P3: “I hope the VR glasses can be made lighter, preferably with adjustable tightness. If I feel dizzy, can the images move slower?”*

*P9: “Can we have disposable protective covers for the glasses, which are replaced after each use? This way, it’s hygienic and we can feel at ease.”*

Content optimization: Focus on modular, personalized, and lightweight content design to avoid fatigue and memory decline caused by long-term viewing.

*P5: “Divide the content into modules such as environment introduction, instrument explanation, and transfer criteria. I can click on whichever I want to watch first, without having to watch from the beginning to the end.”*

*P12: “If VR can push content according to my type of surgery, focusing on my postoperative care and complication management, it will be more targeted.”*

*P6: “Don’t make me watch for too long at one time; about 10 minutes is enough. After watching, take a break and then watch again, otherwise I won’t remember it either.”*

Support system optimization: Focus on the dual needs of operational guidance and emotional support.

*P1: “I hope the nurse can stay beside me while I use it. I can ask immediately if I don’t understand something, and the nurse can teach me step by step, instead of letting me figure it out on my own.”*

*P10: “Can my family watch VR with me?” hoping that family accompaniment can relieve tension and improve the effect of information reception with the help of family members’ auxiliary understanding.*

## 4 Discussion

### 4.1 Patients’ positive attitudes toward the use of VR-based preoperative ICU visits

Consistent with the findings of Locke et al. (23), patients generally held a positive willingness to use VR tools, with the main driving factors including curiosity, novelty, the expectation of relieving preoperative anxiety and fear through preoperative visits, and the desire to obtain practical information. In terms of content, patients focused on key information such as the ICU environment and layout, diagnosis and treatment processes, core equipment functions, and transfer criteria. Meanwhile, patients hoped to clarify process details such as communication methods during endotracheal intubation through VR, indicating that the current traditional model relying on verbal notification is obviously unable to meet such refined needs. However, the multi-dimensional presentation of VR scenarios (e.g., simulating the observation of patients’ nursing process from a third-person perspective) can meet their information needs, which provides a practical basis for the development of VR-based preoperative ICU visits.

In terms of function display, patients suggested using more graphics and animations instead of text to improve information transmission efficiency. This may be related to the fact that using graphics rather than text in the user interface can promote more intuitive and efficient information presentation and improve the usability of the VR-based preoperative ICU visits system (24).

### 4.2 Application advantages and value of VR-based preoperative ICU visits

Traditional preoperative visits rely on verbal education and printed materials, which have limitations such as dense professional jargon, lack of ICU pertinence, and no visual support. Medical staff find it difficult to accurately assess patients’ understanding level and efficiently deliver professional knowledge, resulting in limited information transmission effect of verbal education (25, 26). Compared with traditional methods, VR’s ability to provide consistent and engaging educational content makes it a promising tool to improve the experience of patients and providers in healthcare settings (27, 28).

VR can simulate real-world environments by providing users with a sense of presence and creating highly immersive experiences (7). For example, VR can intuitively restore the ICU environment and diagnosis and treatment processes (simulating monitor alarms, endotracheal tube extubation), making up for the defect that traditional verbal education is abstract and difficult to understand (29), and can specifically solve the above problems. Moreover, studies have shown that compared with video education, immersive VR preoperative education is more advantageous in reducing patients’ preoperative anxiety and improving patient compliance (30). Therefore, VR can serve as a valuable supplement to preoperative patient planning (31).

The application value of VR-based preoperative ICU visits is mainly reflected in three aspects: First, relieving anxiety. A study by Yu et al. (17) confirmed that the perioperative anxiety level of patients in the virtual operating room visit group was significantly reduced. Other studies have also shown that VR experience before surgery significantly reduces patients’ preoperative anxiety, pain, and impatience. These beneficial effects may be attributed to familiarity with and exposure to unfamiliar environments through VR education (6, 32). VR can convert abstract medical information into perceivable visual scenarios, helping patients experience unfamiliar environments and diagnosis and treatment processes in advance. In the preoperative context, VR can be used for psychological intervention before patients experience increased clinical distress and suffer from adverse downstream effects of distress, which is similar to stress inoculation training, a form of cognitive-behavioral intervention aimed at preparing mentally for future exposure to stressful environments through initial exposure to elements of the stressful environment (33).

Second, improving information understanding and satisfaction. A prospective randomized controlled trial by Chiu et al. (29) reported that VR-based preoperative education effectively reduced anxiety and information desire in patients waiting for elective surgery and improved their overall satisfaction. Chang et al. (6) reported that patients who received VR-based preoperative education reported significantly higher satisfaction compared with those provided with printed materials. Beyond meeting their needs and delivering accurate medical information, patients reported that VR materials enhanced the effectiveness of education and improved their preparedness for surgery. This highlights the value of VR as an innovative and effective tool for patient education. Third, VR enables the provision of standardized educational content. The quality of education varies depending on the competence of healthcare professionals, and the use of VR for assisted education has the benefit of maintaining consistency (27).

### 4.3 Potential barriers and optimization suggestions for the implementation of VR-based preoperative ICU visits

The core barriers to implementing VR-based preoperative ICU visits fall into four categories: equipment-related barriers (complex operation, wearing discomfort, and hygiene and safety concerns), content-related barriers (information overload and insufficient personalization), patient-related barriers (decline in sensory and cognitive function among older adult patients), and environment-related barriers (lack of on-site guidance and emotional support).

The optimization suggestions proposed by patients were highly targeted: regarding equipment, simplifying the operation interface and equipping disposable protective covers to address concerns about operational difficulty and hygiene; regarding content, adopting modular design and personalized customization to resolve issues of information overload and insufficient targeting; regarding the support system, providing on-site guidance from nurses and allowing family accompaniment to meet both operational needs and emotional support. These suggestions provide a clear and feasible optimization path for the clinical translation of VR-based preoperative ICU visits tools.

Some patients showed a tendency toward low willingness, with their main concerns focusing on the tolerability of VR technology (e.g., dizziness) and the practicality of the content. This is consistent with previous research findings (34), which identified cybersickness and age as potential limiting factors for VR use. A study by Huygelier et al. (35) found that susceptibility to cybersickness decreases with age. In China, the average age of patients admitted to the ICU is relatively high, which may lead to lower susceptibility to cybersickness. Previous similar studies have consistently reported no serious adverse events associated with the use of VR in patient education (17, 29). Naef et al. (36) investigated the optimal duration of visual and auditory stimulation for ICU patients. In their study, visual stimulation should not exceed 10–15 min, while auditory stimulation should not exceed 1 h, to prevent adverse side effects. Therefore, by reasonably controlling the duration of immersive VR experiences (37, 38) and improving the performance of VR devices, the risk of cybersickness can be reduced to a certain extent (39).

Previous studies have reported that older adults may face greater difficulties than younger adults in adopting new technologies, a phenomenon described as the “digital divide,” which stems from differences in self-efficacy and technological confidence, meaning that older patients may be less interested in new technologies (40). However, in an analysis of the correlation between patient age and VR tours, Vogt et al. (41) pointed out that older and younger adults have equally high acceptance of VR technology. Other studies focusing on critically ill ICU populations have also found that even patients with little prior VR experience have high acceptance of immersive VR (23). This facilitates the widespread application of VR technology in ICU preoperative visits.

Nevertheless, during tool development, attention should still be paid to equipment adaptability (e.g., lightweight design) and content presentation (e.g., controlling the speed of scene transitions). Implementation strategies can be optimized by simplifying operational procedures and providing one-on-one guidance to alleviate older patients’ concerns about technical operation. During actual use, close observation is required to provide timely assistance in case of adverse reactions.

Notably, research findings (7) have shown that prior familiarity with VR helps improve the acceptance of postoperative patients admitted to the ICU. The use of VR-based preoperative ICU visits can unlock the potential of VR to provide better services for postoperative patients in the ICU. For example, after ICU admission, patients can use VR for relaxation therapy. If patients are already familiar with the use of VR preoperatively, this can save significant time and effort on introducing and training them on VR later, creating a better environment for the continuous use of VR in the ICU, such as in rehabilitation and pain management (42).

## 5 Innovations, limitations and future directions

### 5.1 Innovations

The innovations of this study are as follows: First, patients’ positive willingness provides a foundation for clinical promotion, supporting the feasibility of subsequent application of VR-based preoperative ICU visits. Second, existing VR tools are mostly designed by medical teams, which may lead to a disconnect between the medical perspective and patient needs. In contrast, this study adopted a patient-centered approach and involved patients in the development process of the VR system, ensuring that the subsequently developed VR-based preoperative ICU visits system can be truly applied to actual clinical users.

### 5.2 Limitations

This study has several limitations. First, single-center sampling was adopted, which may limit the generalizability of the sample. Second, the perspective of medical staff was not included. Due to space constraints and to better present results from different perspectives, the authors plan to report medical staff’s suggestions in a separate article, and integrate them with the findings of this study for the development of the VR-based preoperative ICU visits.

Notably, participants provided feedback based on their conceptual understanding and anticipated use of VR, rather than direct interaction with an implemented system. As such, the findings primarily illuminate anticipated attitudes, perceived suitability, and hypothetical engagement—offering valuable insight into patient perspectives prior to deployment, but not capturing the dynamic realities of actual use. This distinction reflects a common phase in early-stage digital health research, where conceptual feedback serves as a foundational step toward iterative design and future empirical validation.

### 5.3 Future directions

Future research can be advanced in three aspects: First, develop a prototype VR-based preoperative ICU visits tool based on the optimization suggestions from this study, evaluate its feasibility, usability, and actual effects in reducing preoperative anxiety and postoperative delirium, as well as improving cognitive function and cooperation among elective surgery patients through small-scale trials, and conduct iterative optimization. Second, after systematic iteration and optimization, develop a personalized VR-based preoperative ICU visits tool and verify its effects on patient outcomes including preoperative anxiety, postoperative delirium, and length of hospital stay through randomized controlled trials. Third, further explore the integration of VR with other technologies (e.g., artificial intelligence) to achieve intelligent content delivery and real-time interactive feedback, promoting the digital and precise development of perioperative care.

## 6 Conclusion

This qualitative study systematically explored patients’ informational needs, acceptance attitudes, and perceived barriers toward VR-based preoperative ICU visits. Overall, participants showed positive willingness to accept VR intervention, and put forward clear demands for intuitive, scenario-based information covering ICU environment, diagnosis and treatment processes, equipment functions and communication methods during intubation. The limitations of traditional preoperative visits identified in the study align with the immersive and visual features of VR technology, and patients expressed positive attitudes toward the potential application of VR in optimizing preoperative ICU education, though these findings reflect anticipated attitudes toward a hypothetical intervention rather than outcomes from actual VR use.

This study further summarized four dimensions of potential implementation barriers, including equipment, content, patient characteristics, and supporting environment, and proposed corresponding targeted optimization suggestions from the patient perspective. These findings provide patient-centered empirical reference for the design and development of subsequent VR-based preoperative ICU visits tools.

Subsequent research can cooperate with technical teams to construct a VR-based preoperative ICU visits system based on the needs identified in this study. Further clinical studies are warranted to verify the feasibility, usability and clinical efficacy of the intervention, so as to steadily promote the clinical translation and promotion of VR-based preoperative education. In the implementation process, attention should be paid to the use needs of special populations such as older adult patients, and medical institutions may strengthen VR application training for medical staff to provide timely operational guidance and emotional support for patients, to support the realization of the clinical value of VR technology.

## Data Availability

The datasets presented in this article are not readily available because the datasets generated and/or analyzed during the current study are not publicly available due to the qualitative nature of the data and ethical restrictions regarding participant privacy, but are available from the corresponding author on reasonable request. Requests to access the datasets should be directed to liucongcong1988@126.com.

## References

[1] Aydal P Uslu Y Ulus B. The effect of preoperative nursing visit on anxiety and pain level of patients after surgery. J Perianesth Nurs. (2023) 38:96–101. doi: 10.1016/j.jopan.2022.05.086, 35970660

[2] Ramesh C Nayak BS Pai VB Patil NT George A George LS . Effect of preoperative education on postoperative outcomes among patients undergoing cardiac surgery: a systematic review and meta-analysis. J Perianesth Nur. (2017) 32:518–529.e2. doi: 10.1016/j.jopan.2016.11.011, 29157759

[3] Jung MJ Libaw JS Ma K Whitlock EL Feiner JR Sinskey JL. Pediatric distraction on induction of anesthesia with virtual reality and perioperative anxiolysis: a randomized controlled trial. Anesth Analg. (2021) 132:798–806. doi: 10.1213/ane.0000000000005004, 32618627 PMC9387568

[4] Schnock KO Ravindran SS Fladger A Leone K Williams DM Dwyer CL . Identifying information resources for patients in the intensive care unit and their families. Crit Care Nurse. (2017) 37:e10–6. doi: 10.4037/ccn2017961, 29196595

[5] Baytar AD Bollucuo LK. Effect of virtual reality on preoperative anxiety in patients undergoing septorhinoplasty. Braz J Anesthesiol. (2023) 73:159–64. doi: 10.1016/j.bjane.2021.08.014 PMC10068547 34562488

[6] Chang SL Kuo MJ Lin YJ Chen SA Chen CT Yang YY . Virtual reality-based preprocedural education increases preparedness and satisfaction of patients about the catheter ablation of atrial fibrillation. J Chin Med Assoc. (2021) 84:690–7. doi: 10.1097/jcma.0000000000000555, 34029219 PMC12966114

[7] Ju X Jiang L Yang J Zheng Q Liu X. Enhancing patient experience in the surgical icu through virtual reality: a pre-post mixed-methods study. Heart Lung. (2025) 70:93–101. doi: 10.1016/j.hrtlng.2024.11.014, 39631244

[8] Turksal E Alper I Sergin D Yuksel E Ulukaya S. The effects of preoperative anxiety on anesthetic recovery and postoperative pain in patients undergoing donor nephrectomy. Braz J Anesthesiol. (2020) 70:271–7. doi: 10.1016/j.bjan.2020.03.010, 32653228 PMC9373665

[9] Lai VK Lee A Leung P Chiu CH Ho KM Gomersall CD . Patient and family satisfaction levels in the intensive care unit after elective cardiac surgery: study protocol for a randomised controlled trial of a preoperative patient education intervention. BMJ Open. (2016) 6:e011341. doi: 10.1136/bmjopen-2016-011341, 27334883 PMC4932258

[10] Van Rijn MM De Heer LM Nieuwenhuis-Wendt J Van der Kaaij NP Moolenaar EGE Van der Ham DH . Use of virtual reality in preoperative education of cardiac surgery patients – a feasibility study. Patient Educ Couns. (2024) 129:108394. doi: 10.1016/j.pec.2024.108394, 39168039

[11] Yang J Rhu J Lim S Kang D Lee H Choi GS . Impact of virtual reality education on disease-specific knowledge and anxiety for hepatocellular carcinoma patient scheduled for liver resection: a randomized controlled study. Int J Surg. (2024) 110:2810–7. doi: 10.1097/js9.0000000000001197, 38377058 PMC11093422

[12] Wang LJ Casto B Luh JY Wang SJ. Virtual reality-based education for patients undergoing radiation therapy. J Cancer Educ. (2022) 37:694–700. doi: 10.1007/s13187-020-01870-7, 32970303 PMC7512212

[13] Hermans ANL Betz K Verhaert DVM den Uijl DW Clerx K Debie L . 360° virtual reality to improve patient education and reduce anxiety towards atrial fibrillation ablation. Europace. (2023) 25:855–62. doi: 10.1093/europace/euac246, 36738261 PMC10062331

[14] Asoodar M Janesarvatan F Yu H de Jong N. Theoretical foundations and implications of augmented reality, virtual reality, and mixed reality for immersive learning in health professions education. Adv Simul (Lond). (2024) 9:36. doi: 10.1186/s41077-024-00311-5, 39252139 PMC11382381

[15] Stepan K Zeiger J Hanchuk S Del Signore A Shrivastava R Govindaraj S . Immersive virtual reality as a teaching tool for neuroanatomy. Int Forum Allergy Rhinol. (2017) 7:1006–13. doi: 10.1002/alr.21986, 28719062

[16] van der Kruk SR Zielinski R MacDougall H Hughes-Barton D Gunn KM. Virtual reality as a patient education tool in healthcare: a scoping review. Patient Educ Couns. (2022) 105:1928–42. doi: 10.1016/j.pec.2022.02.005, 35168856

[17] Yu Y Zhou X Zeng G Hou Y. Impact of virtual operating room tours on relieving perioperative anxiety in adult patients: a systematic review. J Perianesth Nurs. (2023) 38:657–663.e7. doi: 10.1016/j.jopan.2022.11.013, 36697345

[18] Bui T Ruiz-Cardozo MA Dave HS Barot K Kann MR Joseph K . Virtual, augmented, and mixed reality applications for surgical rehearsal, operative execution, and patient education in spine surgery: a scoping review. Medicina (Kaunas). (2024) 60:332. doi: 10.3390/medicina60020332, 38399619 PMC10890632

[19] Grab M Hundertmark F Thierfelder N Fairchild M Mela P Hagl C . New perspectives in patient education for cardiac surgery using 3d-printing and virtual reality. Front Cardiovasc Med. (2023) 10:1092007. doi: 10.3389/fcvm.2023.1092007, 36937915 PMC10020687

[20] Birckhead B Khalil C Liu X Conovitz S Rizzo A Danovitch I . Recommendations for methodology of virtual reality clinical trials in health care by an international working group: iterative study. JMIR Ment Health. (2019) 6:e11973. doi: 10.2196/11973, 30702436 PMC6374734

[21] Braun V Clarke V. Using thematic analysis in psychology. Qual Res Psychol. (2006) 3:77–101. doi: 10.1191/1478088706qp063oa

[22] Lincoln YS Guba EG. Naturalistic Inquiry. Newbury Park, CA: Sage Publications (1985).

[23] Locke BW Tsai TY Reategui-Rivera CM Gabriel AS Smiley A Finkelstein J. Immersive virtual reality use in medical intensive care: mixed methods feasibility study. JMIR Serious Games. (2024) 12:e62842. doi: 10.2196/62842, 39046869 PMC11344185

[24] Hong S Choi H Kweon H. Medical device based on a virtual reality-based upper limb rehabilitation software: usability evaluation through cognitive walkthrough. JMIR Form Res. (2025) 9:e68149. doi: 10.2196/68149, 40168640 PMC11978233

[25] Xing J Gong C Wu B Li Y Liu L Yang P . Effect of an educational video about eras on reducing preoperative anxiety and promoting recovery. Heliyon. (2023) 9:e20536. doi: 10.1016/j.heliyon.2023.e20536, 37842611 PMC10568319

[26] Palanica A Docktor MJ Lee A Fossat Y. Using mobile virtual reality to enhance medical comprehension and satisfaction in patients and their families. Perspect Med Educ. (2019) 8:123–7. doi: 10.1007/s40037-019-0504-7, 30912006 PMC6468017

[27] Kim J Kim D Oh SH Kwon H. Virtual reality for preoperative patient education: impact on satisfaction, usability, and burnout from the perspective of new nurses. World J Clin Cases. (2024) 12:6204–16. doi: 10.12998/wjcc.v12.i28.6204, 39371559 PMC11362887

[28] Roy M. Enhancing preoperative patient education through virtual reality: a leap forward in nursing practice. World J Clin Cases. (2024) 12:6744–7. doi: 10.12998/wjcc.v12.i34.6744, 39650820 PMC11514351

[29] Chiu PL Li H Yap KY Lam KC Yip PR Wong CL. Virtual reality-based intervention to reduce preoperative anxiety in adults undergoing elective surgery: a randomized clinical trial. JAMA Netw Open. (2023) 6:e2340588. doi: 10.1001/jamanetworkopen.2023.40588, 37906193 PMC10618840

[30] Lee J Ryu JH Kim JH Han SH Park JW. Effects of a virtual reality digital twin of the operating theatre on anxiety in pediatric surgery patients: a randomized controlled trial. Korean J Anesthesiol. (2025) 1:874. doi: 10.4097/kja.24874, 40170591

[31] El Mathari S Kuitert L Boulidam N Shehadeh S Klautz RJM de Lind van Wijngaarden R . Evaluating virtual reality patient education in cardiac surgery: impact on preoperative anxiety and postoperative patient satisfaction. J Clin Med. (2024) 13:567. doi: 10.3390/jcm13216567, 39518707 PMC11546597

[32] Ryu JH Park SJ Park JW Kim JW Yoo HJ Kim TW . Randomized clinical trial of immersive virtual reality tour of the operating theatre in children before anaesthesia. Br J Surg. (2017) 104:1628–33. doi: 10.1002/bjs.10684, 28975600

[33] Sommer JL Reynolds K Hebbard P Smith MSD Mota N Mutch WAC . Preoperative virtual reality to expose patients with breast cancer to the operating room environment: feasibility and pilot case series study. JMIR Form Res. (2024) 8:e46367. doi: 10.2196/46367, 38231570 PMC10831694

[34] Saab MM Hegarty J Murphy D Landers M. Incorporating virtual reality in nurse education: a qualitative study of nursing students' perspectives. Nurse Educ Today. (2021) 105:105045. doi: 10.1016/j.nedt.2021.105045, 34245956

[35] Huygelier H Schraepen B Van Ee R Vanden Abeele V Gillebert CR. Acceptance of immersive head-mounted virtual reality in older adults. Sci Rep. (2019) 9:4519. doi: 10.1038/s41598-019-41200-6, 30872760 PMC6418153

[36] Naef AC Erne K Exl MT Nef T Jeitziner MM. Visual and auditory stimulation for patients in the intensive care unit: a mixed-method study. Intensive Crit Care Nurs. (2022) 73:103306. doi: 10.1016/j.iccn.2022.103306, 35931597

[37] Gao J Liu S Zhang S Wang Y Liang Z Feng Q . Pilot study of a virtual reality educational intervention for radiotherapy patients prior to initiating treatment. J Cancer Educ. (2022) 37:578–85. doi: 10.1007/s13187-020-01848-5, 32829456

[38] Aardoom JJ Hilt AD Woudenberg T Chavannes NH Atsma DE. A preoperative virtual reality app for patients scheduled for cardiac catheterization: pre-post questionnaire study examining feasibility, usability, and acceptability. JMIR Cardio. (2022) 6:e29473. doi: 10.2196/29473, 35191839 PMC8905473

[39] Cipresso P Giglioli IAC Raya MA Riva G. The past, present, and future of virtual and augmented reality research: a network and cluster analysis of the literature. Front Psychol. (2018) 9:2086. doi: 10.3389/fpsyg.2018.02086, 30459681 PMC6232426

[40] Mosadeghi S Reid MW Martinez B Rosen BT Spiegel BM. Feasibility of an immersive virtual reality intervention for hospitalized patients: an observational cohort study. JMIR Ment Health. (2016) 3:e28. doi: 10.2196/mental.5801, 27349654 PMC4940605

[41] Vogt L Klasen M Rossaint R Goeretz U Ebus P Sopka S. Virtual reality tour to reduce perioperative anxiety in an operating setting before anesthesia: randomized clinical trial. J Med Internet Res. (2021) 23:e28018. doi: 10.2196/28018, 34252034 PMC8444035

[42] Lai VKW Ho KM Wong WT Leung P Gomersall CD Underwood MJ . Effect of preoperative education and icu tour on patient and family satisfaction and anxiety in the intensive care unit after elective cardiac surgery: a randomised controlled trial. BMJ Qual Saf. (2021) 30:228–35. doi: 10.1136/bmjqs-2019-010667, 32321777

