# Supplementary material for: Patients’ willingness and needs for ICU virtual reality visits before elective surgery: a qualitative study
Source: Front Public Health. 2026 Sep 9;14:1902729. doi: 10.3389/fpubh.2026.1902729 (PMC13597700; doi:10.3389/fpubh.2026.1902729)
Supplement: Supplementary file 1 [file Table_1.docx]

**Consolidated criteria for reporting qualitative studies (COREQ): 32-item checklist**

| **No.** | **Item** | **Description** | **Section #** |
| --- | --- | --- | --- |
| **Domain 1: Research team and reflexivity** | | | |
| Personal characteristics | | | |
| *1.* | Interviewer/facilitator | Which author/s conducted the interview or focus group? | 2.2.2 Data Collection |
| *2.* | Credentials | What were the researcher's credentials? *E.g. PhD, MD* | 2.2.2 Data Collection |
| *3.* | Occupation | What was their occupation at the time of the study? | 2.2.2 Data Collection |
| *4.* | Gender | Was the researcher male or female? | 2.2.2 Data Collection |
| *5.* | Experience and training | What experience or training did the researcher have? | 2.2.2 Data Collection |
| Relationship with participants | | | |
| *6.* | Relationship  established | Was a relationship established prior to study commencement? | 2.2.2 Data Collection |
| *7.* | Participant knowledge of the interviewer | What did the participants know about the researcher? *E.g. Personal goals, reasons for doing the research* | 2.2.2 Data Collection |
| *8.* | Interviewer  characteristics | What characteristics were reported about the interviewer/facilitator? *E.g. Bias, assumptions, reasons and interests in the research topic* | 2.2.2 Data Collection |
| **Domain 2: Study design** | | | |
| Theoretical framework | | | |
| *9.* | Methodological  orientation and theory | What methodological orientation was stated to underpin the study? *E.g. grounded theory,*  *discourse analysis, ethnography,*  *phenomenology, content analysis* | 2.2.3. Data Processing and Analysis |
| Participant selection | | | |
| *10.* | Sampling | How were participants selected? *E.g. purposive, convenience, consecutive, snowball* | 2.1. Participants |
| *11.* | Method of approach | How were participants approached? *E.g. face- to-face, telephone, mail, email* | 2.2.2. Data Collection |
| *12.* | Sample size | How many participants were in the study? | 2.1. Participants |
| *13.* | Non-participation | How many people refused to participate or  dropped out? What were the reasons for this? | 2.2.2 Data Collection |
| Setting | | | |
| *14.* | Setting of data collection | Where was the data collected? *E.g. home, clinic, workplace* | 2.2.2 Data Collection |
| *15.* | Presence of non- participants | Was anyone else present besides the participants and researchers? | 2.2.2 Data Collection |

| *16.* | Description of sample | What are the important characteristics of the sample? *E.g. demographic data, date* | 2.1. Participants |
| --- | --- | --- | --- |
| Data collection | | | |
| *17.* | Interview guide | Were questions, prompts, guides provided by the authors? Was it pilot tested? | 2.2.1. Development of the Interview Guide |
| *18.* | Repeat interviews | Were repeat interviews carried out? If yes, how many? | N/A |
| *19.* | Audio/visual recording | Did the research use audio or visual recording to collect the data? | 2.2.2 Data Collection |
| *20.* | Field notes | Were field notes made during and/or after the interview or focus group? | 2.2.2 Data Collection |
| *21.* | Duration | What was the duration of the interviews or focus group? | 2.2.2 Data Collection |
| *22.* | Data saturation | Was data saturation discussed? | 2.2.2 Data Collection |
| *23.* | Transcripts returned | Were transcripts returned to participants for comment and/or correction? | 2.2.3. Data Processing and Analysis |
| **Domain 3: analysis and findings** | | | |
| Data analysis | | | |
| *24.* | Number of data coders | How many data coders coded the data? | 2.2.3. Data Processing and Analysis |
| *25.* | Description of the coding tree | Did authors provide a description of the coding tree? | Table 2. Thematic Coding Examples |
| *26.* | Derivation of themes | Were themes identified in advance or derived from the data? | 2.2.3. Data Processing and Analysis |
| *27.* | Software | What software, if applicable, was used to manage the data? | 2.2.3. Data Processing and Analysis |
| *28.* | Participant checking | Did participants provide feedback on the findings? | 2.2.3. Data Processing and Analysis |
| Reporting | | | |
| *29.* | Quotations presented | Were participant quotations presented to  illustrate the themes / findings? Was each  quotation identified? *E.g. Participant number* | 3. Results |
| *30.* | Data and findings consistent | Was there consistency between the data presented and the findings? | 3. Results  4. Discussion |
| *31.* | Clarity of major themes | Were major themes clearly presented in the findings? | 3. Results |
| *32.* | Clarity of minor themes | Is there a description of diverse cases or discussion of minor themes? | 3. Results |

Developed from: Allison Tong, Peter Sainsbury, Jonathan Craig, Consolidated criteria for reporting qualitative research

(COREQ): a 32-item checklist for interviews and focus groups, International Journal for Quality in Health Care, Volume 19,

Issue 6, December 2007, Pages 349-357,<https://doi.org/10.1093/intqhc/mzm042>
